# Predication of the Effector Proteins Secreted by *Fusarium sacchari* Using Genomic Analysis and Heterogenous Expression

**DOI:** 10.3390/jof8010059

**Published:** 2022-01-06

**Authors:** Zhen Huang, Huixue Li, Yuming Zhou, Yixue Bao, Zhenzhen Duan, Caixia Wang, Charles A. Powell, Baoshan Chen, Muqing Zhang, Wei Yao

**Affiliations:** 1 State Key Lab for Conservation and Utilization of Subtropical Agri-Biological Resources, Guangxi Key Lab of Sugarcane Biology, Guangxi University, Nanning 530005, China; huangzhen@st.gxu.edu.cn (Z.H.); 1817301010@st.gxu.edu.cn (H.L.); 1817301042@st.gxu.edu.cn (Y.Z.); byx2020@gxu.edu.cn (Y.B.); dzz2017@gxu.edu.cn (Z.D.); 2717301037@st.gxu.edu.cn (C.W.); chenyaoj@gxu.edu.cn (B.C.); 2 IRREC-IFAS, University of Florida, Fort Pierce, FL 34945, USA; capowell@ufl.edu

**Keywords:** sugarcane, *Fusarium sacchari*, effector proteins, PCD, qRT-PCR

## Abstract

One of the causative agents of pokkah boeng disease (PBD), which affects sugarcane crops globally, is the fungus *Fusarium sacchari*. These fungal infections reduce sugar quality and yield, resulting in severe economic losses. Effector proteins play important roles in the interactions between pathogenic fungi and plants. Here, we used bioinformatic prediction approaches to identify 316 candidate secreted effector proteins (CSEPs) in the complete genome of *F. sacchari*. In total, 95 CSEPs contained known conserved structures, representing 40 superfamilies and 18 domains, while an additional 91 CSEPs contained seven known motifs. Of the 130 CSEPs containing no known domains or motifs, 14 contained one of four novel motifs. A heterogeneous expression system in *Nicotiana benthamiana* was used to investigate the functions of 163 CSEPs. Seven CSEPs suppressed BAX-triggered programmed cell death in *N. benthamiana*, while four caused cell death in *N. benthamiana*. The expression profiles of these eleven CSEPs during *F. sacchari* infection suggested that they may be involved in sugarcane-*F. sacchari* interaction. Our results establish a basis for further studies of the role of effector molecules in pathogen–sugarcane interactions, and provide a framework for future predictions of pathogen effector molecules.

## 1. Introduction

Sugarcane (*Saccharum* spp.) is the most important sugar crop worldwide, producing approximately 80% of all sugar worldwide [1], as well as more than 90% of all sugar in China [2]. Pokkah boeng disease (PBD), which is caused by the *Fusarium fujikuroi* species complex (FFSC), is one of the most common and serious fungal diseases of sugarcane [3]. FFSC leads to significant yield losses in susceptible varieties of sugarcane worldwide [3,4]. *F. sacchari*, a species in the FFSC, is tightly associated with both PBD and stalk wilt in sugarcane. *F. sacchari* infections thus severely affect sugarcane yield and productivity [5,6]. Meng et al. found that *F. sacchari* was primarily a causative agent of PBD during the summer, as hot, humid conditions prompt this species to produce large numbers of conidia, thus increasing the likelihood of infection [7]. In order to develop new and improved PBD control strategies, it is necessary to better understand the mechanisms underlying the interactions between *F. sacchari* and its hosts.

During plant–pathogen interactions, plants recognize pathogen-related molecular patterns (PAMPs) and damage-associated molecular patterns (DAMPs) using plasma membrane-localized pattern recognition receptors (PRRs), thus initiating the first-line host defense system known as PAMP-triggered immunity (PTI) [8,9]. Host-adapted pathogens express avirulence (Avr) genes and release “effector” substances into host cells, which interfere with the PTI response or enhance pathogen virulence [10,11]. In response, plants have co-evolved effector-specific resistance proteins, encoded by host-resistant genes (R genes), that combine directly with pathogen effector proteins or indirectly monitor effector-triggered perturbations in host proteins [11,12,13]. This second-line defense process, known as effector-triggered immunity (ETI), concurrently activates a stronger defense response and is usually accompanied by localized tissue necrosis (the hypersensitive response) [8].

Fungal effector proteins can be roughly grouped based on their mode of deployment within the host: extracellular effectors are secreted into the apoplast or xylem of the host plant, while cytoplasmic effectors are translocated into host cells [14,15]. The precise details of effector function remain unclear. However, examples from biotrophic, hemi-biotrophic, and necrotrophic pathogens show that effector proteins both benefit and hinder pathogenic invasion [16]. Effector proteins increase host susceptibility via multiple pathways, especially by suppressing plant defense responses [17]. For example, effector proteins interfere with the recognition of microbe-related molecules by the PRRs, block intracellular signal transduction, and suppress the expression of R genes, thereby promoting pathogen infection, expansion, and colonization [9,14,18,19]. Conversely, effector proteins are recognized by PRRs and nucleotide-binding domain and leucine-rich repeat receptors (NLRs), and thus act as immune-system signals that induce plant defense responses [20]. Therefore, pathogen effector identification is critical for the development of crop resistance [16]. An improved understanding of fungal-effector function and the associated underlying mechanisms, in conjunction with the use of host-induced gene silencing technology to produce disease-resistant crops, may represent a critical strategy for the prevention and control of plant diseases in the future [21].

Since the first fungal Avr gene was cloned (from *Cladosporium fulvum*) in 1991 [22], several fungal effectors from pathogens associated with serious plant diseases have been characterized and investigated. Unfortunately, due to the low degree of sequence conservation among the vast majority of fungal effectors as compared to those of bacteria and oomycetes, the identification of fungal effector proteins is particularly challenging [23]. As whole-genome sequencing technology has matured, and as the number of sequenced pathogen genomes has increased, the computational prediction of effector proteins has become a viable, rapid, and economical method by which to identify candidate secreted effector proteins (CSEPs) for subsequent experimental validation [24]. Comprehensive analyses of known effectors have identified several features that accurately predict CSEPs in fungi [25,26,27,28]: (i) having an N-terminal signal peptide; (ii) lacking transmembrane domains; (iii) lacking glycosyl-phosphatidyl-inositol (GPI)-anchor sites; (iv) lacking a predicted location signal for protein delivery to mitochondria or other intracellular organelles; and (v) comprising about 50–300 amino acid residues. Fungal CSEPs also generally exhibit a number of shared secondary features, including cysteine-richness, a high degree of sequence specificity, and similar locations in the host plant [28]. Based on these characters, several pathogenic fungal effector proteins have been predicted in rice, wheat, corn, and other crops using computational methods. However, although predicting effector proteins using software is an efficient strategy, experimental verification is necessary to explore the specific functions of fungal growth, infection, colonization and interaction with plants.

To date, little is known about the pathogenic mechanisms of *F. sacchari*. Here, we used several computational prediction methods to identify CSEPs in the whole genome of *F. sacchari*. After analysis and characterization, the CSEPs were expressed in *Nicotiana benthamiana* leaves using *Agrobacterium tumefaciens*-mediated transient gene expression to investigate gene function. We also used quantitative real-time PCR (qRT-PCR) to characterize gene expression patterns during infection. Our results establish a framework for the identification of additional effector proteins from *F. sacchari*, subsequently helping to clarify the molecular mechanisms underlying the interactions between sugarcane and its pathogens.

## 2. Materials and Methods

### 2.1. Materials

The *Fusarium sacchari* strain used in this study was obtained from laboratory cultures and grown on potato dextrose agar (PDA) at 28 °C in the dark. Sugarcane cultivar “ZZ-1” is susceptible to *F. sacchari*. Healthy, mature sugarcane stalks with uniform growth rates were selected from our fields and cultured in a barrel in a greenhouse. Sugarcane seedlings were inoculated with *F. sacchari* at the 5-leaf stage. *N. benthamiana* plants, which were used for transient gene expression, were grown in a growth chamber under a 16/8 h light/dark cycle at 25 °C with 60% humidity. *Escherichia coli* Top10 competent cells, which were used to propagate plasmids, and *A. tumefaciens* strain GV3101 (pJIC SA_Rep), which was used for *A. tumefaciens*-mediated transient gene expression, were purchased from Shanghai Weidi Biotechnology Co., Ltd. (Shanghai, China). The potato virus X (pGR106) vector used in this study was generously provided by Prof. Zhensheng Kang (Northwest A&F University, Xianyang, China).

### 2.2. Prediction of Fungal CSEPs

The complete genome of *F. sacchari* was previously obtained by our laboratory (unpublished data). An artificial neural network, as implemented in SignalP v4.1 [29], was used to identify N-terminal signal peptides and their cleavage sites as previously described [30]. Next, a fasta file containing all retained sequences was constructed. Transmembrane helices were then predicted in these sequences using TMHMM Server V.2.0 with default parameters [31]. We constructed a fasta file containing the retained sequences and submitted this file to the TargetP v1.1 server for subcellular location prediction [32]. The TargetP v1.1 server predicts protein locations based on whether the sequence is predicted to contain any N-terminal presequences: chloroplast transit peptides, mitochondrial targeting peptides, or secretory pathway signal peptides [33]. Finally, we identified all sequences predicted to contain GPI-lipid anchoring modification sites using the big-PI Fungal Predictor server and excluded these ankyrin sequences from further analysis [34].

### 2.3. Structural Characters of the CSEPs

The number of cysteine residues in each CSEP was determined using ProtParam (https://web.expasy.org/protparam/, accessed on 7 February 2021). The NCBI Conserved Domain Database was used to determine whether any effector candidates were similar to known conserved domains (https://www.ncbi.nlm.nih.gov, accessed on 9 April 2020) with the cutoff E-value set to 10^−5^. We then used MEGA 7.0 to manually search the CSEPs for amino-acid motifs previously identified as conserved across various plant pathogens [35]. The motifs searched included RXLR, Crinkling and Necrosis (CRN), and CHXC in oomycetes; [Y/F/W]xC in powdery mildew; G[I/F/Y][A/L/S/T]R in flax rust; [L/I]xAR in *Magnaporthe oryzae*; [S/G]PC[K/R]P in various *Fusarium* species; [R/K]VY[L/I]R in *Blumeria gramini*; YxSL[R/K] in *Pythium ultimum*; and the RGD motif in the *Pyrenophora tritici-repentis* ToxA protein [24,28,36,37]. For CSEPs lacking known conserved domains and motifs, we performed de novo structural predictions using MEME (https://meme-suite.org/meme/, accessed on 29 November 2020) [38]. The web version of MEME performs motif discovery on protein datasets in “Classic” mode: site distribution is set to Zero or One Occurrence Per Sequence (zoops).

### 2.4. Plasmid Construction and Preparation

Exogenous genes can be transiently expressed in *N. benthamiana* to determine whether these genes participate in pathogenic infection [39,40]. The pGR106 vector is often used to express target genes in *N. benthamiana*. Primers were designed based on the complementary DNA (cDNA) sequence containing the longest open reading frame (ORF). Total RNA was extracted from the *F. sacchari* strain using a TaKaRa MiniBEST Universal RNA Extraction Kit (Takara, Beijing, China). Based on preliminary results, we cloned CSEPs from the cDNA of *F. sacchari* using 2× Phanta Max Master Mix (Vazyme, Nanjing, China). Using the ClaI-SmaI-SalI restriction enzyme cutting site, the PCR products were double digested and ligated with the previously double-digested vector pGR106 using restriction enzymes and T4 ligase (Takara, Beijing, China). *E. coli* Top10 cells were transformed with the ligation product following the instructions. The sequence of the resulting plasmid was verified using PCR and DNA sequencing by Sangon Biotech (Shanghai, China). The primers used for plasmid construction are listed in Supplementary Table S1.

### 2.5. Transient Expression of Target CSEPs in N. benthamiana

The recombinant plasmids and the pGR106 vector were transformed into *A. tumefaciens* strain GV3101 (pJIC SA_Rep) using the freeze–thaw method [41]. GV3101 cultures containing the target recombinant constructs were cultured for 48 h at 28 °C with shaking at 220 rpm in Luria-Bertani (LB) broth containing 50 mg/mL Kanamycin, 20 mg/mL Rifampicin, and 50 mg/mL Gentamicin. Transformant cells were harvested by centrifugation at 4000 rpm for 4 min at room temperature and washed three times with 10 mM MgCl2. Before infiltration, bacterial suspensions were adjusted to an optical density at 600 nm (OD600) of 0.5 and incubated for 1 h in the dark. We used four- to six-week-old *N. benthamiana* plants for the agroinfiltration assays. Aliquots of the bacterial suspensions were infiltrated into *N. benthamiana* leaves using a needleless syringe [40]. To test the suppression of cell death, *N. benthamiana* leaves were infiltrated with an *A. tumefaciens* strain carrying a plasmid harboring BAX 24 h after infiltration with the *A. tumefaciens* carrying a plasmid harboring a CSEP. *A. tumefaciens* cells carrying pGR106-BAX and empty vector pGR106 were used as positive and negative controls, respectively. Each assay was replicated using at least 25 leaves across six plants.

### 2.6. Verification of the Secretory Function of the Signal Peptide

The functional validation of the predicted signal peptides of the 11 effector proteins was performed using the yeast signal sequence trap system as described in a previous study [42]. The recombinant pSUC2 vector constructs were transformed into *Saccharomyces cerevisiae* strain YTK12, and the transformants were grown on CMD-W (lacking tryptophan) media comprised of 0.67% yeast N base without amino acids, 0.075% tryptophan dropout supplement, 2% sucrose, 0.1% glucose, and 2% agar. Clones were identified using PCR with vector-specific primers (Table S1). For invertase secretion testing, positive clones were incubated on YPRAA media comprised of 1% yeast extract, 2% peptone, 2% raffinose, and 2 μg/mL antimycin A. YTK12 cells transformed with pSUC2-Avr1bSP were used as the positive control, while the empty vectors pSUC2 and mg87SP were used as negative controls, following the protocols of a previous study [43].

### 2.7. qRT-PCR Validation of Target Genes

*F. sacchari* isolates were cultured on a PDA plate for seven days. Fungal blocks were punched at the edges of activated *F. sacchari* colonies using a hole punch. We pricked both sides of the middle vein of each sugarcane leaf and pressed the mycelium side of the fungal block. To encourage infection, we kept the pricked site wet for 24 h. Leaf samples were collected at 0, 12, 24, 48, 72, 96, 120, 168 h, and 216 h post-inoculation (hpi); three biological replicates were collected at each time point. Three replicate mycelium samples were collected for comparison. Total RNA was extracted from the leaf and mycelium samples using TaKaRa MiniBEST Universal RNA Extraction Kits (Takara, Beijing, China), following the manufacturer’s instructions. Total RNA (500 ng) extracted from each sample was reverse transcribed into cDNA using oligo (dT) primers and a PrimeScript RT reagent Kit (Perfect Real Time) (Takara, Beijing, China), following the manufacturer’s instructions. cDNA was amplified using a fast two-step amplification program with TB Green Premix Ex Taq II (Tli RNaseH Plus) (Takara, Beijing, China) on a LightCycler 96 system (Roche, Mannheim, Germany). The *F. verticillioides* actin (*ACT1*) gene was used as the internal control against which to normalize gene expression levels in each sample [44]. Relative gene expression was quantified using the 2^−ΔΔCt^ method [45]. Three technical replicates were analyzed per sample. Means and standard deviations were calculated, and figures were drawn, using GraphPad Prism 8.2.1 (GraphPad Software, La Jolla, CA, USA). The primers were designed with Primer Premier 5.0 (Premier Biosoft, Palo Alto, CA, USA), and the NCBI Primer-BLAST tool (https://www.ncbi.nlm.nih.gov/tools/primer-blast/, accessed on 19 June 2019) was used to verify primer specificity (Table S1).

## 3. Results

### 3.1. Comprehensive CSEP Prediction

**Figure 1:**
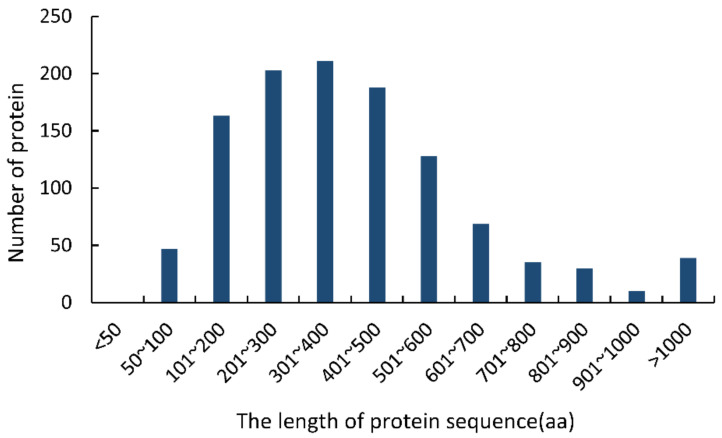
Lengths of the proteins containing N-terminal signal peptides in the *Fusarium sacchari* genome.

The following typical characters were used to identify CSEPs in the *F. sacchari* genome [25,26,27,28]: (i) having N-terminal signal peptides; (ii) consisting of 50–300 amino acid residues; (iii) lacking transmembrane domains; (iv) lacking GPI-anchor sites; and (v) lacking predicted location signals for protein delivery to mitochondria or other intracellular organelles (Supplementary Figure S1). Initially, 1124 sequences containing N-terminal signal peptides were identified in the *F. sacchari* genome. Of these, 36.78% (413 sequences) were 50–300 aa long; none of the sequences were less than 50 aa long (Figure 1). TMHMM analysis showed that 373 of the 413 sequences (50–300 aa) lacked transmembrane domains. Of these, 364 sequences were predicted to contain extracellularly excreted signal peptides with high confidence, and 316 of those sequences were shown to lack GPI anchor sites. These 316 protein sequences were thus considered final CSEPs.

### 3.2. The CSEPs of F. sacchari Had Typical Structural Characteristics

**Figure 2:**
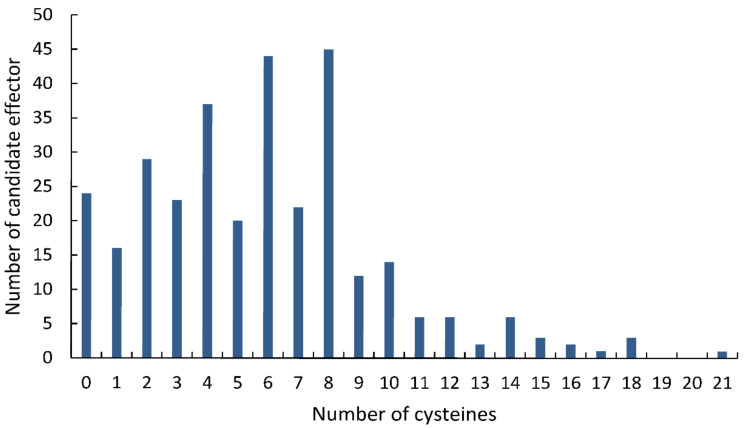
Distribution of cysteines across the candidate secreted effector proteins identified in the *Fusarium sacchari* genome.

Statistical analysis showed that 286 (90.50%) of the 316 final CSEPs contained 10 or fewer cysteines, while 224 CSEPs (70.88%) had at least four cysteine residues (Figure 2). Most of the CSEPs contained eight cysteines (45 CSEPs), followed by six cysteines (44 CSEPs), four cysteines (37 CSEPs), and two cysteines (29 CSEPs). Only 24 CSEPs lacked cysteines entirely. Notably, one CSEP (*Fs*12526) contained 21 cysteines.

**Figure 3:**
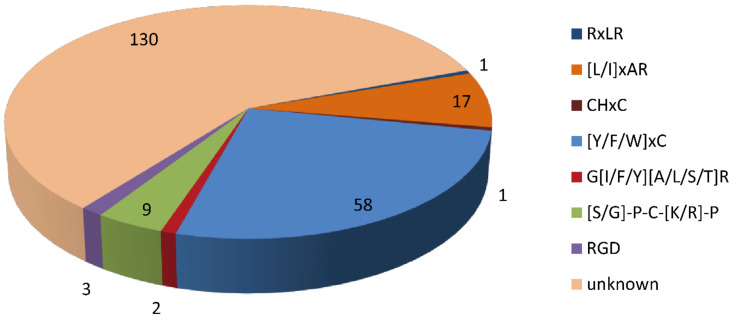
Distribution of known and unknown motifs across the 221 candidate secreted effector proteins lacking conserved domains.

**Figure 4:**
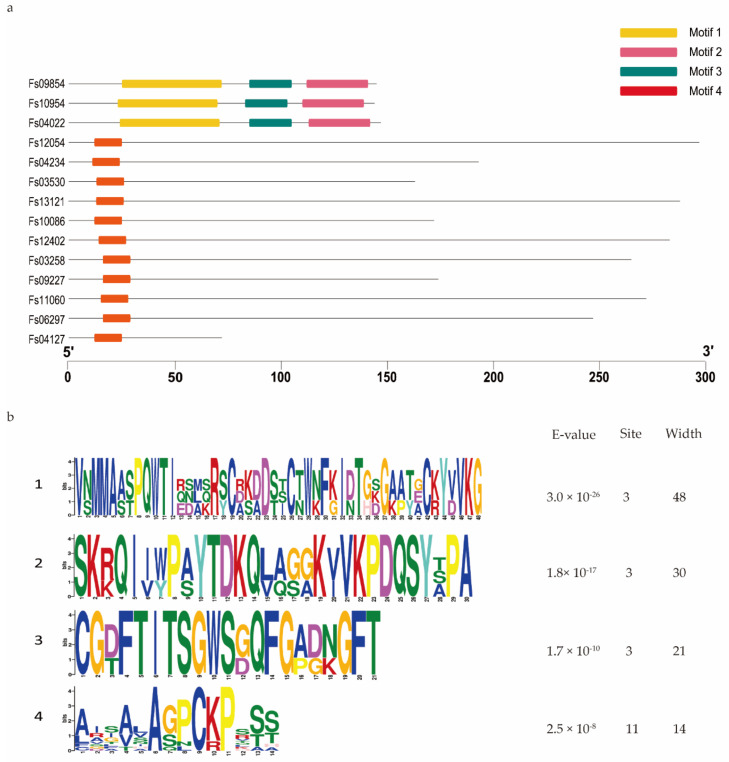
De novo prediction of four new motifs in the 130 candidate secreted effector proteins lacking both conserved domains and conserved motifs. (**a**) Four motifs were predicted based on the 130 candidate secreted effector proteins, using MEME. (**b**) Details of the four motifs.

Domain analysis showed that 95 of the 316 CSEPs contained known conserved structures, representing 40 protein superfamilies and 18 conserved domains (Table 1). Manual searching of the remaining 221 CSEPs sequences without known conserved domains identified a total of seven distinct motifs (Figure 3): 58 of these CSEPs (26.24%) harbored the powdery mildew [Y/F/W]xC motif, 17 CSEPs harbored the *Magnaporthe oryzae* [L/I]xAR motif, 9 CSEPs harbored the *Fusarium* [S/G]PC[K/R]P motif, 3 CSEPs harbored the *P. tritici*-*repentis* RGD motif, and 2 CSEPs harbored the flax rust G[I/F/Y][A/L/S/T]R motif. The oomycete RXLR and CHXC motifs were each found in one CSEP. The remaining 130 CSEPs did not contain any known motifs. De novo prediction of the motifs in these 130 CSEP sequences identified four distinct novel motifs across 14 CSEPs (E-value > 10^−5^; Figure 4). Three distinct motifs were present in the same three CSEPs (*Fs*09854, *Fs*10954, and *Fs*04022); a fourth motif was present in 11 CSEPs (Figure 4).

### 3.3. Certain CSEPs Induced PCD or Suppressed BAX-Triggered PCD in N. benthamiana

**Figure 5:**
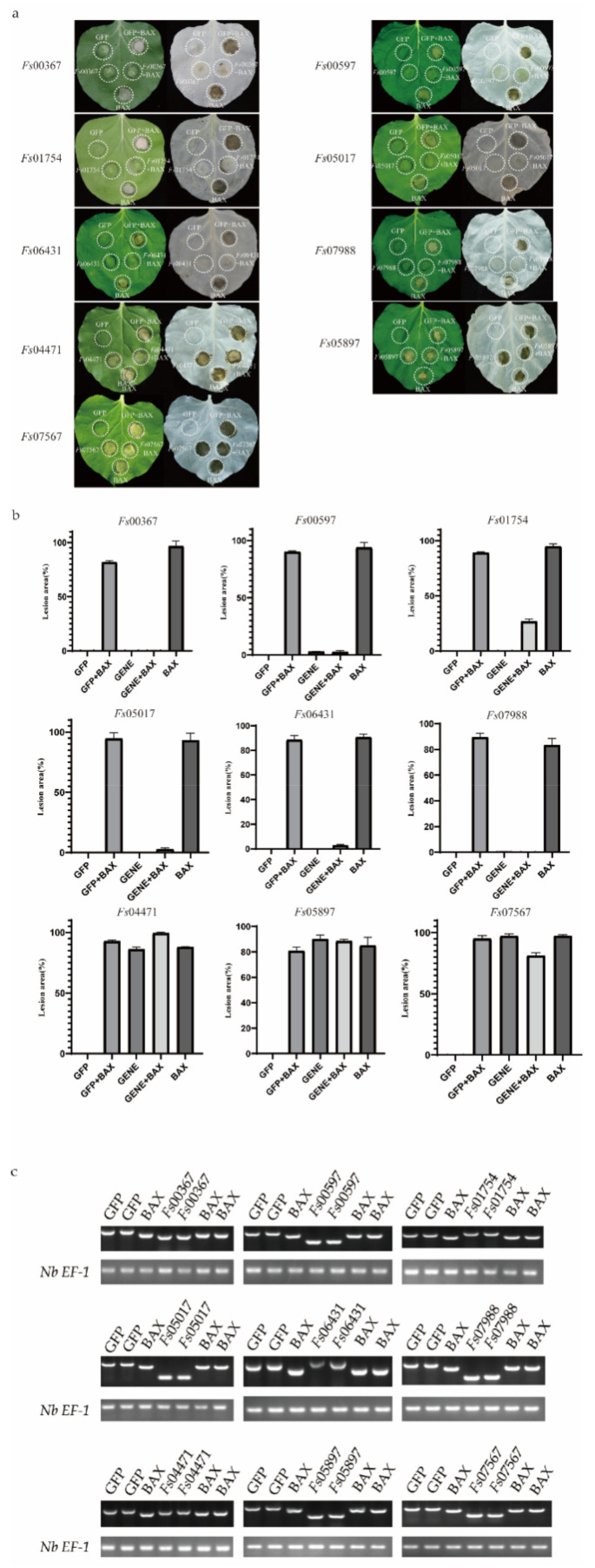
Transient expression of 9 candidate secreted effector proteins (CSEPs) from *Fusarium sacchari* in *Nicotiana* *benthamiana* leaves. (**a**) Transient expression of candidate secreted effector proteins (CSEPs) in *N. benthamiana*. *N. benthamiana* leaves were infiltrated with *A. tumefaciens* cells containing PVX vectors carrying green fluorescent protein (GFP) (negative control) or CSEPs. At 24 h after infection, *A. tumefaciens* cells carrying the pGR106-BAX vector were infiltrated. Photos were taken 7 days after infiltration. Cells were decolorized using ethanol for ease of visualization. Each assay was replicated using at least 25 leaves across six plants. (**b**) The area of each lesion was calculated using ImageJ [46]. Means and standard errors were calculated from three independent experiments. (**c**) Total RNA was extracted from the leaves of 2-days infected tobacco, RT-PCR was performed to identify the gene expression using the cDNA from the effected tobacco leaves as templates; the *Nb EF-1* [43] was used as the reference gene.

To explore the roles of the candidate secreted effector proteins in plant immunity, we successfully isolated 230 genes from the cloned complementary DNA (cDNA). However, we were unable to construct the recombinant vector for 13 genes because the genes had two or three restriction enzyme cutting sites. Vector construction failed for an additional 54 genes. In total, 163 recombinant vectors were obtained. The *A. tumefaciens*-mediated transient expression of the CSEPs in *N. benthamiana* was performed, with GFP and BAX serving as negative and positive controls, respectively. The CSEPs *Fs*00367, *Fs*00597, *Fs*01754, *Fs*05017, *Fs*07988, and *Fs*06431 suppressed BAX-induced cell death, while, similar to BAX, *Fs*04471, *Fs*05897, and *Fs*07567 induced cell death (Figure 5a). The areas of necrosis were more obvious after the decolorization of tobacco leaves with ethanol. The area of each lesion was determined using ImageJ (Figure 5b) [46].

### 3.4. Validation of the Signal Peptides of Candidate Effector Proteins

**Figure 6:**
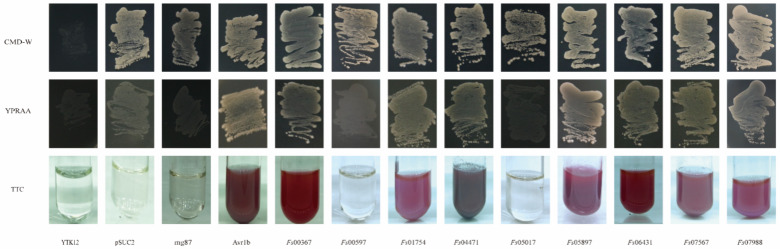
Functional validation of the CSEP signal peptides using yeast invertase secretion assays. The signal peptide was fused into the pSUC2 vector and transformed into the yeast YTK12 strain. The predicted signal peptide of pSUC2-Avr1b was used as a positive control. Non-transformed YTK12, YTK12 carrying the pSUC2 vector, and YTK12 carrying the pSUC2-Mg87 vector were used as negative controls. Yeast growth on CMD-W (lacking Trp) medium confirmed that the vector was transformed into the yeast strain, while growth on YPRAA medium and TTC color change confirmed invertase secretion.

The yeast mutant YTK12, which lacks the sucrose invertase gene, failed to grow on CMD-W medium (containing sucrose and glucose, but not tryptophan). Although the pSUC2 plasmid contains the tryptophan synthesis gene, this plasmid lacked a signal peptide and the ATG initiation codon for the sucrose invertase gene. Therefore, yeast transformants harboring the pSUC2 plasmid grew naturally in the CMD-W medium but not in the YPRAA medium containing raffinose only. The reduction of triphenyltetrazolium chloride (TTC) to insoluble red 1,3,5-triphenyltetrazolium was monitored to detect secreted invertase activity [47]. All 11 candidate effector proteins contained signal peptides at the N-terminal, suggesting that these proteins had potential secretory functions. These putative secretory functions were verified using a yeast invertase secretion assay for the signal peptides. The strains transformed with the pSUC2 vector, which was used as a negative control, grew on the CMD-W medium, but not on the YPRAA medium. In contrast, strains carrying the Avr1b signal peptide, which was used as the positive control, grew on both CMD-W and YPRAA media. The signal peptides of *Fs*00367, *Fs*01754, *Fs*04471, *Fs*05897, *Fs*06431, *Fs*07567, and *Fs*07988 rescued the defect in the sucrose invertase gene of YTK12, allowing this strain to secrete invertase. If the signal peptide exhibited secretory functions, YTK12, carrying the recombinant vector pSUC2, would grow on the YPRAA medium with raffinose as the sole carbon source. Additionally, a TTC color reaction was performed to determine if the predicted signal peptides had secretory functions. Fructosidase SUC2 was secreted into the extracellular domain, reducing 2,3,5-TTC to red 1,3,5-triphenyltetrazolium (Figure 6). These results suggested that these seven proteins (*Fs*00367, *Fs*01754, *Fs*04471, *Fs*05897, *Fs*06431, *Fs*07567 and *Fs*07988) were typical secretory proteins; *Fs*00597 and *Fs*05017 may have unique secretory pathways. In our previous studies, yeast invertase secretion assays using the signal peptides of *Fs*00548 and *Fs*11724 confirmed that these proteins had secretory functions [43,48].

### 3.5. CSEP Expression Profiles in F. sacchari at Different Stages of Infection

**Figure 7:**
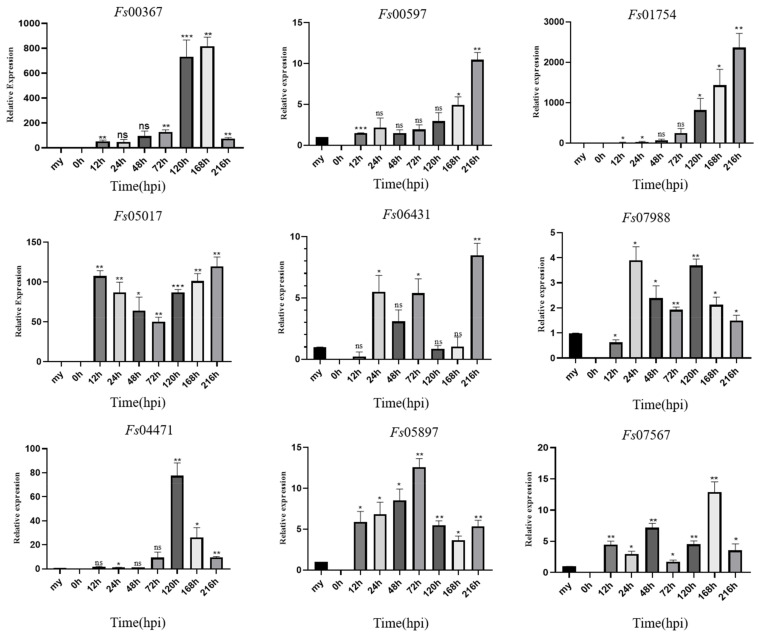
Expression profiles of nine candidate effector proteins from *Fusarium sacchari* during infection of sugarcane leaves as compared to baseline expression in mycelia (my). Relative gene expression was normalized against the expression of endogenous actin gene. Error bars represent the standard deviations of the means of three technical and biological replicates. Asterisks indicate significant differences in gene expression level relative to the mycelia baseline: * *p* < 0.1, ** *p* < 0.05, *** *p* < 0.01, ns = no significant difference; Statistical significance was determined using two-way ANOVAs.

During the infection of sugarcane cultivar ZZ-1 by *F. sacchari*, qRT-PCR analysis showed that the 11 CSEPs analyzed tended to become increasingly upregulated with respect to expression levels in the mycelia as the infection progressed (Figure 7). Specifically, *Fs*04471 was significantly upregulated between 72 and 120 hpi, peaking at 120 hpi, and becoming increasingly downregulated after 168 hpi. *Fs*05897 was continuously expressed from 12 hpi onwards, with expression increasing between 12 and 72 hpi, peaking at 72 hpi, and then decreasing. *Fs*07988 was continuously expressed from 24 hpi onwards, with two induced expression peaks: one at 24 h and one at 120 h. Both *Fs*00597 and *Fs*01754 were steadily upregulated as the infection progressed, peaking at 216 hpi. Notably, *Fs*00367 was expressed during all stages of infection but was most strongly upregulated at 168 hpi. *Fs*01754 was extremely strongly upregulated at 216 hpi, with a ~2300-fold increase in relative expression over that in the mycelia. *Fs*06431 was also expressed from 24 hpi onwards, with expression decreasing at 120 h, but subsequently increased to a peak at 216 hpi.

## 4. Discussion

Secreted effector proteins are critical for pathogenic fungal invasion because they manipulate host processes to support efficient colonization [16,19,49]. Due to the increasing accessibility of *genome*-wide *sequencing* technologies, various pathogen genomes are now available. Using these genomes, effector proteins with various functions have been identified in a variety of pathogens, including *Puccinia striiformis* f. sp. *tritici (Pst)*, *Cladosporium fulvum*, *Ustilago maydis*, *Magnaporthe oryzae,* and *Phytophthora capsici* [50,51,52,53,54]. Due to the lack of conserved features across fungal effector protein sequences, fungal effector prediction approaches are based on relatively broad criteria, principally the presence of a secretion signal [14,55]. Secreted proteins can be divided into two general categories: classical secreted proteins and non-classical secreted proteins. The few proteins secreted via non-classical pathways lack conventional signal peptides and are not dependent on the membrane secretion system of the endoplasmic reticulum and the Golgi apparatus [55]. Here, we primarily focused on proteins secreted via classical pathways, and we predicted secreted proteins based on their N-terminal signal peptides.

The software tools we used to predict CSEPs have previously been described [23]. Based on the results of these bioinformatics analyses, we obtained 316 CSEPs. Similar results were reported in *Beauveria bassiana* [56]. We then constructed a candidate effector protein library to facilitate our subsequent exploration of the role of pathogenic effector proteins in plant immunity.

Cysteine content is frequently used to identify candidate apoplastic effector proteins, as many of the cysteines present in fungi likely form the intramolecular disulfide bonds required for stability and function in the protease-rich apoplast [15]. Our results showed that most (82.9%) of the 316 CSEPs contained between 1 and 10 cysteines, with 4, 6, and 8 cysteines being the most common. Similar cysteine patterns have been observed in the CSEPs of other pathogens, including *Phytophthora cinnamomic* [57] and *F. graminearum* [58]. However, we found that the 11 effector proteins identified herein differed with respect to length and the cysteine content. For example, *Fs*06431 had 15 cysteines, while *Fs*05897 lacked cysteines entirely. Despite these differences in cysteine content, all 11 effector proteins showed the ability to induce or suppress plant immunity. Thus, cysteine enrichment cannot be used as the sole criterion by which to identify effectors. However, cysteine enrichment can serve as an important reference for experimental analyses of effector function.

The phytopathological functions of some CSEPs have previously been identified. Based on a Pfam domain search, in conjunction with the identification of conserved domains, we identified several proteins that contained conserved domains and motifs. Our analysis indicated that the functions of only 40 of the superfamilies identified in the CSEPs had previously been characterized. One of the common families identified in the CSEPs was the hydrolase family. Proteins in the hydrolase family have been confirmed to be effectors [59,60]. These proteins are involved in fungal growth and development processes, including spore germination [61], hyphal growth and branching emergence [62], and basidiomycete fruiting and development [63]. The pathogenesis-related 1 proteins (CAP) superfamily, is important for sterol binding and export, as well as fungal virulence [64]. However, the molecular mode of action of this protein family has remained enigmatic [65]. Finally, the lysin motif (LysM) is a widely distributed protein motif that binds to (peptido) glycans [66]. Fungal effectors containing a LysM domain mediate virulence by perturbing chitin-triggered host immunity [67]. Such effectors include extracellular protein 6 (Ecp6), secreted by *Cladosporium fulvum* [68]; secreted LysM protein 1 (Spl2), secreted by *Magnaporthe oryzae* [69], and Mg1LysM and Mg3LysM, secreted by *Mycosphaerella graminicola* [70].

We identified 18 conserved domains across the 95 CSEPs harboring known structures. Of these, carbohydrate-binding modules are found in multidomain proteins carrying functionally related modules [59]. Similar multidomain proteins carrying the peptidoglycan domain and the chitin-binding domain have been identified in other phytopathogenic fungi effectors [69,71,72]. For example, the Ecp2 effector protein of the tomato pathogen *Cladopsorium fulvum* carries the Hce2 domain, which is thought to induce necrosis in plants and increase fungal pathogenicity [73,74,75]. The necrosis-inducing *Phytophthora* protein 1 (NPP1) domain, which was first obtained from *Phytophthora parasitica*, induces hypersensitive cell death-like lesions in parsley [76]. In addition, NPP1 structural homologs, such as the Nep1-like proteins, have been identified in bacteria, oomycetes, and fungi [77], while the cerato-platanin fungal domain has been shown to act as an elicitor [78]. Pectate lyases have also been shown to play an important role in pathogenicity and the induction of plant immunity [79,80]. These findings help to clarify fungus–plant interactions. Several other domains, including peroxidase, fungal hydrophobin, and hydrophobic surface binding protein (HsbA), were also identified in the CSEPs. Thus, although some of the superfamilies and domains identified in the CSEPs are known to participate in plant–fungus interactions, the biological functions of most of the conserved domains remain to be characterized.

The structures and functions of the CSEPs lacking known domains were difficult to predict [23]. However, we found that several CSEPs contained conserved motifs. Fungal or oomycete pathogen effectors containing these conserved motifs are considered “core effectors”, and play crucial roles during pathogenic infection [81]. Interestingly, 58 *F. sacchari* CSEPs contained the [Y/F/W]xC motif, similar to the number of CSEPs carrying the [Y/F/W]xC motif in wheat leaf rust fungus (57 CSEPs) [82]. In barley powdery mildew fungus, all CSEPs possess an N-terminal [Y/F/W]xC motif within 30 amino acids of the signal peptide [82]. This sequence is predicted to fold into a structure similar to that of ribonucleases [83]. The [Y/F/W]xC motif was also found in some wheat leaf rust fungus CSEPs, but with less positional conservation [84]. The [L/I]xAR motif was originally identified based on its conservation across the effector proteins of *Hyaloperonospora arabidopsidis*, *Phytophthora infestans*, and *Phytophthora sojae* [28]. Here, 17 CSEPs harbored the [L/I]xAR motif, consistent with results in *Magnaporthe oryzae* [85]. No RXLR motifs have been identified in fungal effectors [86]. The conserved [S/G]PC[K/R]P motif is located immediately after the signal peptide in several proteins from various *Fusarium* species [87]. Consistent with this, nine CSEPs carrying the [S/G]PC[K/R]P motif were identified in *F. sacchari*. Interestingly, although the C-terminal RGD sequence motif in the ToxA effector is required for wheat cell entry [88], only three RGD motifs were identified across all *F. sacchari* CSEPs. The oomycete motifs RXLR and CHXC were each found in one *F. sacchari* CSEP; no CRN motifs were found in this study. Thus, *F. sacchari* CSEPs exhibit some sequence homology with known fungal effector protein motifs. However, as most of these motifs have yet to be functionally characterized, it is difficult to predict the function of *F. sacchari* effectors solely based on the conserved motifs of other pathogens. Further experimental evidence is required.

We identified four distinct motifs, unique to *F. sacchari* CSEPs, in 14 of the 130 CSEPs that contained no known domains or motifs. Interestingly, part of one of these unique motifs (motif 4) overlapped with the [S/G]PC[K/R]P motif in various *Fusarium species*, suggesting that this motif may act as an important pathogenic effector. However, the functions of these novel motifs remain to be confirmed experimentally.

Many effectors that regulate plant immunity in various pathogens have been systematically identified and characterized [89,90,91]. Several such studies have performed transient expression assays using *N. benthamiana* to preliminarily screen putative effectors that may suppress or induce cell death [92]. For example, most of the 169 avirulence homolog (Avh) proteins identified in the *Phytophthora sojae* genome were shown to suppress BAX-triggered programmed cell death in *N. benthamiana* after *A. tumefaciens*-mediated transient expression [93]. Similarly, more than half of 50 putative effector proteins randomly selected from the genome of the fungus *Ustilaginoidea virens* suppressed the *Burkholderia glumae*-triggered hypersensitive reaction in *N. benthamiana* [94]. Conversely, 11 of 169 *Phytophthora sojae* effectors triggered cell death in *N. benthamiana* leaves [93], while four *Magnaporthe oryzae* effectors induced cell death in *N. benthamiana* when they contained a signal peptide [95]. Although *N. benthamiana* is not a natural host of *F. sacchari*, numerous studies of other pathogen effectors have shown that these effectors possess the same ability to suppress or induce cell death in both non-host and host plants. Indeed, we found that seven CSEPs suppressed BAX-induced cell death and four CSEPs induced cell death in *N. benthamiana.* This indicated that immune-related functions may vary across the 11 CSEPs. However, the interactions between pathogens and plant immune systems require further experimental exploration. For example, techniques such as BioID, gene deletion, host-induced gene silencing (HIGS), yeast two-hybrid system (Y2H), bimolecular fluorescence complementation (BiFC), and immunoprecipitation may be used to explore the interaction of effectors and host targets.

Several previous studies have primarily aimed to identify the signal peptides of effector proteins [43,96], with yeast secretion systems used to confirm secretory function [97]. In general, secreted proteins are required for successful disease development and to determine host–pathogen compatibility [98]. Here, the signal peptides of *Fs*00367, *Fs*00548 [43], *Fs*01754, *Fs*04471, *Fs*05897, *Fs*06431, *Fs*07567, *Fs*07988, and *Fs*11724 [48] were shown to have secretory activity, indicating that these CSEPs were secretory proteins. As *Fs*00597 and *Fs*05017 were not classical secretory proteins, this result hints at the involvement of other secretory pathways.

The expression patterns of many CSEPs from various pathogens have previously been characterized [28]. Here, CSEP production and expression varied among the stages of infection. For example, *Fs*00367, *Fs*00597, and *Fs*01754 were all gradually upregulated as infection progressed, similar to the previously reported results in *U. maydis* and *Zymoseptoria tritici* [99,100]. However, *Fs*01754 was more strongly upregulated than *Fs*00367 and *Fs*00597, suggesting that *Fs*01754 plays a more critical role than either *Fs*00367 or *Fs*00597. The expression patterns of the other eight CSEPs differed from those of *Fs*00367, *Fs*00597 and *Fs*01754. *Fs*05017 and *Fs*06431 were most highly expressed at 216 hpi. The expression levels of *Fs*07988 and *Fs*04471 peaked at 24 hpi and 120 hpi, respectively, and *Fs*05897 and *Fs*00548 peaked at 72 hpi. *Fs*00367, *Fs*07567 and *Fs*11724 reached their highest expression level at 168 hpi. Across these eight CSEPs, *Fs*05017 and *Fs*04471 were the most strongly upregulated, suggesting that these CSEPs may play more important roles in pathogen colonization. Following a previous study, fungal spores were swollen at 24 hpi and mycelium spread to the trichomes at 72 hpi [101]. Compared with the cytology of the infection process [101], *Fs*05017 and *Fs*07988 had high expression from 0–24 hpi, indicating that they may help fungal spores swell. *Fs*00548 and *Fs*05897 peaked at 72 hpi, suggesting they may participate in mycelium extension. *Fs*04471 expression level peaked at 120 hpi, while the expression levels of *Fs*00367, *Fs*07567 and *Fs*11724 peaked at 168 hpi, indicating that these genes may be associated with fungal colonization. *Fs*00597, *Fs*01754 and *Fs*06431 were mainly expressed at 216 hpi and therefore they may inhibit BAX–induced cell death and be involved in suppressing the plant immunity. Overall, the differences in expression patterns across these CSEPs suggest that these proteins are indeed fungal effector proteins, but that their functions and associated mechanisms differ. Thus, these CSEPs are likely to play different roles in the pathogenic process.

## Figures and Tables

**Table 1:** Numbers of unique conserved domains harbored by the 316 candidate secreted effector proteins identified in the *Fusarium sacchari* genome.

	Description	Number
Superfamily	alpha_CA superfamily	2
	CAP superfamily	3
	SGNH_hydrolase superfamily	7
	FkpA superfamily	1
	cupin_like superfamily	2
	ML superfamily	1
	ZnMc superfamily	3
	DUF1961 superfamily	1
	LysM superfamily	2
	Cupredoxin superfamily	2
	PRK11907 superfamily	1
	CE4_SF superfamily	2
	Glyco_hydro_12 superfamily	1
	SurE superfamily	1
	GAT_1 superfamily	2
	CHRD superfamily	1
	DOMON_like superfamily	1
	VOC superfamily	1
	LamG superfamily	3
	Trypsin superfamily	1
	microbial_RNases superfamily	1
	Glyco_hydro_114 superfamily	1
	Abhydrolase superfamily	3
	MhpC superfamily	1
	RNase_T2 superfamily	2
	DUF3455 superfamily	3
	YdcF-like superfamily	1
	Fasciclin superfamily	1
	PLN00052 superfamily	1
	YoaJ superfamily	2
	Hydrophobin superfamily	1
	SodA superfamily	1
	DPBB_1 superfamily	1
	M35_like superfamily	1
	cysteine_hydrolases superfamily	1
	CM_2 superfamily	1
	Cupin_5 superfamily	1
	Lyz_like superfamily	1
	DUF3237 superfamily	1
	Fimbrial superfamily	1
Domain	CVNH	2
	Hce2	2
	LicD	1
	Pectate_lyase	4
	NPP1	3
	Glyco_hydro_61	4
	Cerato-platanin	3
	Cutinase	6
	mannanase_GH134	1
	PAN_1	1
	Glyco_hydro_11	4
	Methyltransf_23	1
	EthD	2
	TenA_C_Bt3146-like	1
	Hydrophobin_2	2
	CBM_4_9	1
	TNT	1
	WSC	1
	Peroxidase_2	1
	HsbA	1

## Data Availability

The data that support the findings of this study are available from the corresponding author upon reasonable request.

## Supplementary Materials

The following are available online at https://www.mdpi.com/article/10.3390/jof8010059/s1, Table S1: Primers used in this study. Figure S1: Pipeline used to identify candidate secreted effector proteins (CSEPs) in the *Fusarium sacchari* genome. Table S2: The conserved domain contained by the 11 candidate secreted effector proteins.
