# Supplementary figures and images for: Predication of the Effector Proteins Secreted by Fusarium sacchari Using Genomic Analysis and Heterogenous Expression
Source: J Fungi (Basel). 2022 Jan 6;8(1):59. doi: 10.3390/jof8010059 (PMC8780550; doi:10.3390/jof8010059)

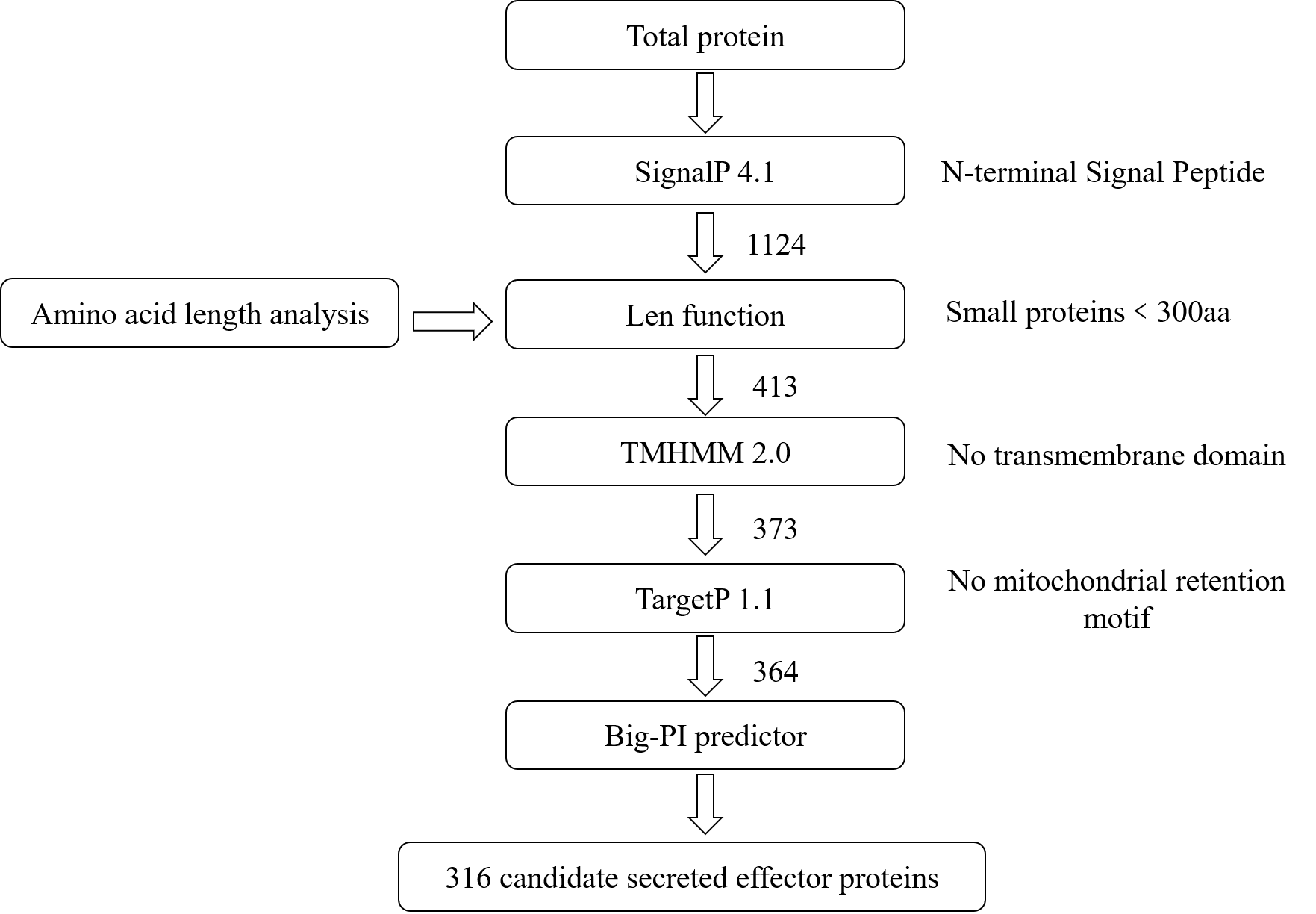

Supplement: Supplementary file 1 [file jof-08-00059-s001.zip › jof-1497915-supplementary/Supplementary/Supplementary Figure S1.tif]
